# Effect of the Plasticizer-to-Polymer Ratio on the Properties of In Situ-Prepared PCL/PEG-Based Polyurethane Elastomers

**DOI:** 10.3390/polym18192402

**Published:** 2026-10-01

**Authors:** Tianqi Li, Kaixuan Feng, Fengling Wang, Yunjun Luo

**Affiliations:** School of Materials Science & Engineering, Beijing Institute of Technology, Beijing 100081, China; litianqi0930@163.com (T.L.); fengkaixuan420@163.com (K.F.); 3120245728@bit.edu.cn (F.W.)

**Keywords:** PCL/PEG-based polyurethane elastomer, in situ preparation, plasticizer-to-polymer ratio, Bu-NENA, microphase structure, thermal decomposition

## Abstract

To investigate the effect of the plasticizer-to-polymer ratio on the structure and properties of in situ-prepared PCL/PEG-based polyurethane elastomers (iPCE elastomers), a series of iPCE elastomers with plasticizer-to-polymer ratios ranging from 0.8 to 1.2 were prepared using Bu-NENA as an energetic plasticizer. Their hydrogen-bonding structure, crosslinked network, segmental mobility, microphase structure, mechanical properties, and thermal decomposition behavior were characterized by Fourier-transform infrared spectroscopy (FTIR), low-field nuclear magnetic resonance (LF-NMR), wide-angle X-ray diffraction (WAXD), small-angle X-ray scattering (SAXS), uniaxial tensile testing, and coupled thermogravimetry–differential scanning calorimetry–Fourier-transform infrared spectroscopy (TG-DSC-FTIR). The results showed that, as the plasticizer-to-polymer ratio increased from 0.8 to 1.2, the fraction of hydrogen-bonded carbonyl groups decreased from 50.11% to 41.62%, exhibiting a strong negative linear correlation with the plasticizer-to-polymer ratio. Increasing the plasticizer-to-polymer ratio prolonged the transverse relaxation times, decreased the apparent crosslink density, and increased the average molecular weight between crosslinks, indicating a progressive reduction in the effective constraints imposed by the network on segmental motion. No distinct crystalline diffraction peaks were observed for any of the elastomers, whereas microphase separation was evident at all plasticizer-to-polymer ratios. The characteristic domain spacing increased from 4.39 to 4.65 nm, indicating an expansion of the characteristic periodicity of the microphase-separated structure. Owing to the combined effects of weakened hydrogen-bond association, reduced apparent crosslink density, and diminished interchain interactions, the tensile strength, elongation at break, and toughness decreased from 2.29 MPa, 166.80%, and 2.16 MJ·m^−3^ to 1.09 MPa, 62.32%, and 0.39 MJ·m^−3^, respectively. The Bu-NENA-containing iPCE elastomers underwent three principal mass-loss stages, corresponding to the decomposition of Bu-NENA, degradation of the polyurethane network, and further decomposition of the residual structures at elevated temperatures. The mass-loss fraction in each stage was broadly consistent with the relative proportions of Bu-NENA and polyurethane components. The plasticizer-to-polymer ratio primarily altered the relative mass loss associated with each stage, while exerting only a limited influence on the characteristic temperatures and composition of the principal decomposition stages.

## 1. Introduction

Energetic plasticizers are commonly incorporated into highly filled composite solid propellants to reduce slurry viscosity, facilitate mixing and casting, and minimize the adverse effects of conventional inert plasticizers on energetic performance [1,2,3,4,5]. Both the type and content of the plasticizer can affect not only the rheological properties of solid propellants but also their mechanical properties, thermal decomposition behavior, and combustion characteristics [6,7,8,9]. In polymeric binder systems, physically incorporated plasticizers can increase the spacing between polymer chains, facilitate segmental motion, and weaken interchain interactions [10,11,12,13]. In chemically crosslinked polyurethanes, the resulting changes in segmental mobility and intermolecular interactions may further affect hydrogen-bonding associations, physical network constraints, and microphase-separation characteristics, thereby altering the macroscopic mechanical response of the elastomers [14,15,16,17]. 

N-Butyl-N-(2-nitroxyethyl) nitramine (Bu-NENA) is an energetic plasticizer characterized by low viscosity, a low glass-transition temperature, favorable energetic performance, and relatively low mechanical sensitivity compared with conventional nitrate ester plasticizers [18,19,20,21]. Previous studies have investigated the compatibility of Bu-NENA with energetic polymers, its interactions with polyurethane binders, and its effects on the mechanical and thermal properties of binders and propellants [22,23]. Ghoroghchian et al. [11] reported that Bu-NENA exhibited favorable compatibility with a PPG-GAP-PPG triblock copolymer and reduced both the glass-transition temperature and viscosity of the system. Fu et al. [12] employed molecular dynamics simulations to investigate the interactions of HTPE with Bu-NENA and other energetic plasticizers. Wang et al. [24] compared the effects of Bu-NENA and BDNPA/F on the properties of binders for high-energy nitrate ester plasticized polyether propellants. In a subsequent study, Wang et al. [25] compared Bu-NENA with geminal dinitro ester plasticizers and further demonstrated that the molecular structure of the plasticizer affects binder viscosity, glass-transition temperature, thermal decomposition behavior, and the energetic performance of the formulation. Yuan and Luo [14] investigated the hydrogen-bonding state, crosslinked-network characteristics, and mechanical properties of HTPE/Bu-NENA binders over a range of plasticizer-to-polymer ratios. Their results showed that increasing the Bu-NENA content weakened hydrogen-bonding associations and network constraints, accompanied by a deterioration in mechanical properties. These studies demonstrate that the Bu-NENA content is an important factor governing the network structure and mechanical response of polyether-based polyurethane binders.

The plasticizing effect of Bu-NENA also depends on the chemical composition and segmental structure of the polyurethane matrix. Previous studies of HTPE/Bu-NENA binders have focused primarily on polyether-based polyurethane systems, whereas the PCL/PEG-based polyurethane investigated in the present work contains both polyester- and polyether-derived soft segments. Previous studies have employed PCL and PEG to construct various polyurethane elastomers and have shown that the PCL/PEG composition affects the segmental structure, phase characteristics, and mechanical properties of the resulting polyurethanes [26,27]. In other polyurethanes containing PCL-based mixed soft segments, the soft-segment composition and sequence have also been shown to affect hydrogen bonding, microphase structure, and mechanical response [28]. PCL diol introduces ester-containing soft segments into the polyurethane network, whereas PEG contributes polyether soft segments. Consequently, the present system differs from HTPE-based polyurethane in terms of carbonyl composition, segmental structure, and intermolecular interactions. Microphase separation in polyurethane is governed primarily by the thermodynamic compatibility between the soft and hard segments, hydrogen-bonding interactions, and segmental structure, and is closely related to the mechanical properties of the material [17]. Meanwhile, externally added plasticizers can alter intermolecular interactions and effective network constraints in polyurethane, although the extent of these effects depends on the chemical composition and network structure of the polyurethane matrix [29]. The effects of Bu-NENA on this mixed-soft-segment polyurethane therefore cannot be fully inferred from observations obtained for HTPE/Bu-NENA systems. For the PCL/PEG-based system, the extent to which changes in carbonyl hydrogen bonding, segmental mobility, and apparent network parameters are accompanied by changes in the characteristic length scale of the microphase-separated structure as the Bu-NENA content increases remains insufficiently understood. The relationships of these structural changes with the load-bearing capacity and deformation stability of the elastomer also require further clarification. Previous work using DSC-TG-MS-FTIR showed that inert and energetic plasticizers can affect the thermal decomposition processes and evolved gaseous products of hydroxyl-terminated polyether-based polyurethanes [30]. However, the effects of the Bu-NENA-to-polymer ratio on the relative mass loss, characteristic temperature, and evolved gaseous products associated with each decomposition stage of PCL/PEG-based polyurethane remain to be established.

In this study, a series of in situ-prepared PCL/PEG-based polyurethane elastomers, hereafter referred to as iPCE elastomers, were prepared with Bu-NENA-to-polymer mass ratios ranging from 0.8 to 1.2, while the PCL/PEG composition, IPDI/N100 composition, and NCO/OH molar ratio were maintained constant. Fourier-transform infrared spectroscopy (FTIR) was used to characterize the carbonyl hydrogen-bonding state, and low-field nuclear magnetic resonance (LF-NMR) was employed to analyze segmental mobility and apparent network parameters. Wide-angle X-ray diffraction (WAXD), small-angle X-ray scattering (SAXS), and uniaxial tensile testing were further used to examine changes in microphase-separation characteristics and mechanical properties. Coupled thermogravimetry–differential scanning calorimetry–Fourier-transform infrared spectroscopy (TG-DSC-FTIR) was employed to analyze the thermal decomposition stages, thermal effects, and evolved gaseous products of the elastomers with different plasticizer-to-polymer ratios. The objective of this study was to determine how the Bu-NENA-to-polymer ratio affects carbonyl hydrogen bonding, segmental mobility, effective network constraints, and microphase-separation characteristics in PCL/PEG-based polyurethane elastomers and to clarify the relationships of these structural changes with their mechanical properties and thermal decomposition behavior.

## 2. Experimental

### 2.1. Materials

Poly (ethylene glycol) (PEG; *M_n_* ≈ 200 g·mol^−1^, nominal functionality = 2) and polycaprolactone diol (PCL; *M_n_* ≈ 1000 g·mol^−1^, nominal functionality = 2) were supplied by Shanghai Macklin Biochemical Co., Ltd. (Shanghai, China) and Juren Chemical New Material Technology Co., Ltd. (Yueyang, China), respectively. Isophorone diisocyanate (IPDI) was purchased from Shanghai Merrier Biochemical Technology Co., Ltd. (Shanghai, China). The polyfunctional isocyanate N100 (*M_n_* ≈ 725 g·mol^−1^, average functionality = 3.87) and N-butyl-N-(2-nitroxyethyl)nitramine (Bu-NENA) were provided by the Liming Research Institute of Chemical Industry, Henan, China. The molar NCO/OH ratio was fixed at 1.0 for all polyurethane formulations. The quantities of the raw materials used for representative formulation with a plasticizer-to-polymer ratio of 1.0 are listed in Table 1.

### 2.2. Preparation

Prior to formulation, PCL and PEG were dehydrated separately under vacuum (ZYMC-180V) for 48 h at 120 and 90 °C, respectively. The required quantities of PCL, PEG, Bu-NENA, and IPDI were then combined according to the designed stoichiometric ratio and manually mixed for 10 min. N100 and the catalyst were subsequently incorporated, and the formulation was mixed thoroughly until visually homogeneous. Entrapped air was initially removed by homogenization for 5 min, followed by vacuum degassing for 10 min. The degassed mixture was carefully transferred into a polytetrafluoroethylene (PTFE) mold with internal dimensions of 15 cm × 7 cm × 1 cm and maintained at 60 °C in a thermostatically controlled water bath for 7 days to complete curing. After demolding, the samples were conditioned at room temperature for 7 days and subsequently cut into dumbbell-shaped specimens for mechanical testing using a cutting die.

### 2.3. Characterizations

#### 2.3.1. Mechanical Testing

Uniaxial tensile tests were performed using an AGS-J universal testing machine (Shimadzu, Kyoto, Japan) at 25 °C in accordance with GB/T 528 [31]. The crosshead speed was set to 100 mm·min^−1^. The narrow parallel section of each dumbbell-shaped specimen was 12 mm long and 2 mm wide. Five independent specimens were tested for each formulation, and the results are reported as the mean ± standard deviation. A schematic showing the dimensions of the narrow section of the specimen has been included in the Supplementary Materials as Figure S1.

#### 2.3.2. Crosslink Density Measurements

For LF-NMR measurements, the elastomer samples were cut into approximately 2 mm × 2 mm pieces and placed in an NMR tube to a packed height of approximately 10 mm. Before each measurement, the samples were equilibrated at the corresponding test temperature for 10 min.

Low-field ^1^H nuclear magnetic resonance measurements were performed using a Bruker Mini spec mq20 spectrometer (Bruker Bio Spin, Rheinstetten, Germany) operating at a proton resonance frequency of 20 MHz Before measurement, the samples were cut into pieces of similar size, placed in NMR tubes, and equilibrated at the measurement temperature for 10 min.

The transverse magnetization decay was recorded using a Carr–Purcell–Mei boom–Gill (CPMG) pulse sequence with an inter-echo time of 0.10 ms, and 36 scans were accumulated for each sample. The *T*_2_ relaxation-time distributions were obtained by applying the CONTIN inverse Laplace transformation to the transverse magnetization decay curves. The T_21_, T_22_, and T_23_ values were determined from the peak positions of the corresponding components in the resulting *T*_2_ distributions.

#### 2.3.3. Fourier-Transform Infrared Spectroscopy

Fourier-transform infrared (FTIR) spectra were recorded using a Nicolet 8700 FTIR spectrometer (Thermo Electron, Waltham, MA, USA) in diffuse-reflectance mode. The spectra were collected over the wavenumber range of 4000–500 cm^−1^ at a resolution of 2 cm^−1^, with 48 scans averaged for each spectrum.

#### 2.3.4. Wide-Angle X-Ray Diffraction

Wide-angle X-ray diffraction (WAXD) patterns were recorded using a D/Max-2500PC X-ray diffractometer (Rigaku, Tokyo, Japan) with Cu Kα radiation (λ = 0.154 nm). The accelerating voltage and tube current were set to 15 kV and 20 mA, respectively, and the scanning rate was 10 °C·min^−1^.

#### 2.3.5. Small-Angle X-Ray Scattering

Small-angle X-ray scattering (SAXS) measurements were performed using a Xeuss 3.0 SAXS/WAXS system equipped with an Eiger2 R 1M detector. Cu Kα radiation with a nominal photon energy of 8.05 keV and a wavelength of 1.54189 Å was used. Elastomer specimens approximately 1.0 mm thick were measured under vacuum (<1 mbar) at a sample-to-detector distance of 70 mm, with an acquisition time of 600 s per sample. The scattering data were analyzed over a q range of 0.2–25 nm^−1^, where the magnitude of the scattering vector was defined as q = 4πsinθ/λ, with 2θ denoting the scattering angle. The background scattering recorded from an empty sample holder under identical conditions was subtracted from the sample scattering data, which were subsequently normalized to the sample transmission.

#### 2.3.6. TG-DSC-FTIR Analysis

Coupled thermogravimetry–differential scanning calorimetry–Fourier-transform infrared spectroscopy (TG-DSC-FTIR) analysis was conducted using an STA 449 F3 Jupiter simultaneous thermal analyzer (NETZSCH, Selb, Germany) coupled with a Tensor 27 FTIR spectrometer (Bruker, Germany). Approximately 5–10 mg of each sample was placed in an alumina crucible and heated from room temperature to 600 °C at a heating rate of 10 °C·min^−1^ under a high-purity nitrogen atmosphere flowing at 50 mL·min^−1^. The evolved gases were transferred to the FTIR gas cell through a heated transfer line. Both the transfer line and gas cell were maintained at 200 °C to minimize condensation. The FTIR spectra of the evolved gases were collected over the range of 4000–400 cm^−1^ at a resolution of 4 cm^−1^.

## 3. Results and Discussion

### 3.1. Effect of the Plasticizer-to-Polymer Ratio on the Structural Characteristics of iPCE Elastomers

#### 3.1.1. Hydrogen-Bonding Interactions

**Figure 1:**
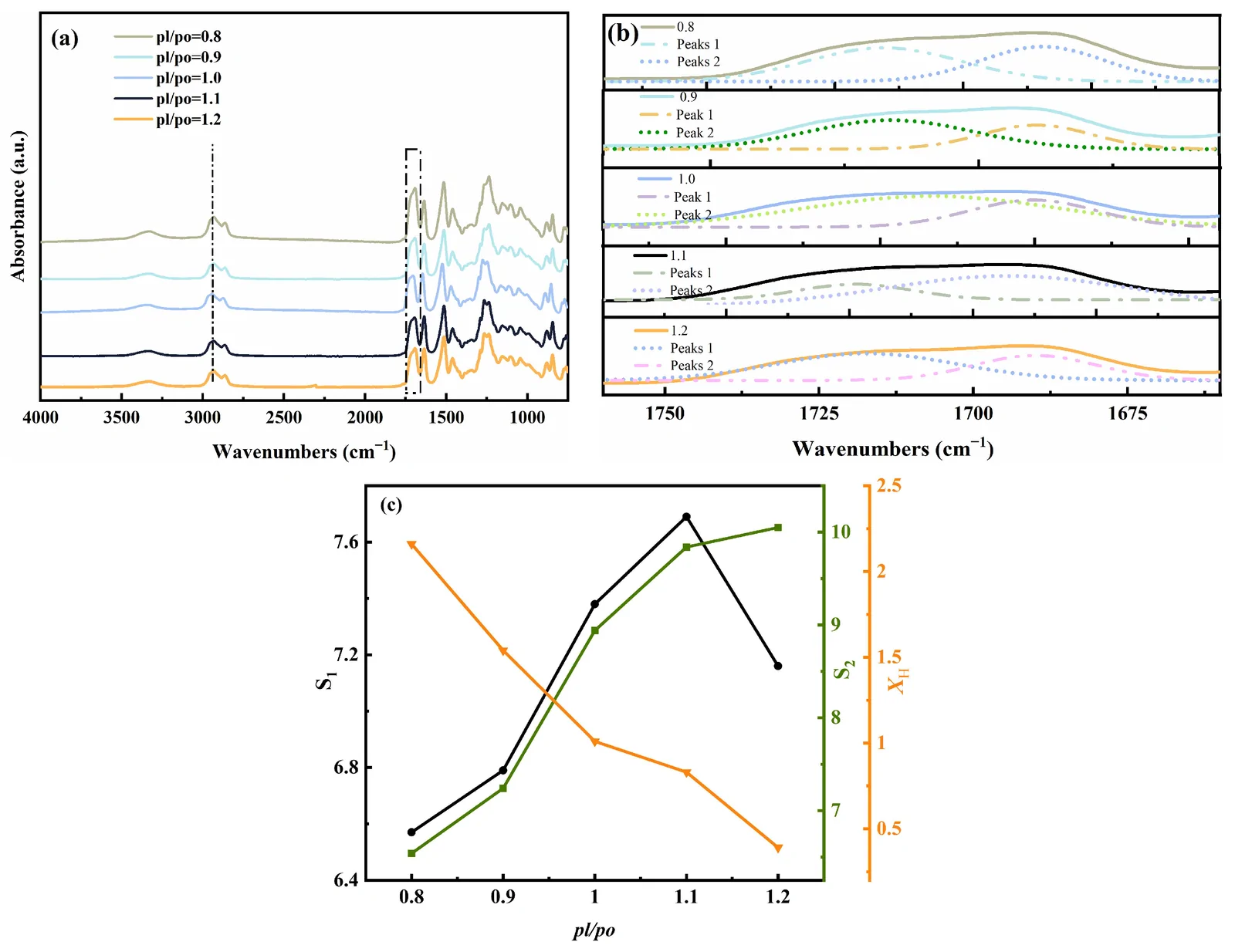
FTIR analysis of iPCE elastomers with different plasticizer-to-polymer ratios: (**a**) FTIR spectra; (**b**) deconvoluted carbonyl region; and (**c**) integrated peak areas and fraction of hydrogen-bonded carbonyl groups.

Hydrogen bonding plays an important role in hard-segment association, microphase structure, and segmental mobility in polyurethane elastomers, thereby affecting their mechanical and thermal properties. The hydrogen-bonding structure of the iPCE elastomers prepared at different plasticizer-to-polymer ratios was therefore characterized by FTIR spectroscopy. The resulting spectra are presented in Figure 1a.

The carbonyl region (1650–1760 cm^−1^) was deconvoluted using Origin software (2025) after linear baseline correction. Two Gaussian functions were employed, with initial peak positions set at approximately 1690 and 1725 cm^−1^, corresponding to hydrogen-bonded and free carbonyl groups, respectively. During nonlinear least-squares fitting, the peak positions, widths, and intensities were allowed to vary within the selected spectral region. Iteration continued until convergence was achieved. The fitting quality was evaluated using the coefficient of determination and the distribution of residuals, with no evident systematic deviation in the residual curve. The area of each carbonyl band was determined by integrating the corresponding fitted Gaussian function. The fraction of hydrogen-bonded carbonyl groups (*X_H_*) was calculated using Equation (1):

*X_H_* = A_H_/(A_H_ + A_F_) × 100% (1)

where A_H_ and A_F_ represent the integrated areas of the fitted bands assigned to hydrogen-bonded and free carbonyl groups, respectively.

The urethane carbonyl region was deconvoluted to distinguish the contributions from hydrogen-bonded and free carbonyl groups. The corresponding peak areas were obtained from the fitted spectra and used to calculate the fraction of hydrogen-bonded carbonyl groups. The fitting results are shown in Figure 1b, and the calculated fractions are summarized in Table 1.

As shown in Table 1, the fraction of hydrogen-bonded carbonyl groups decreased progressively with increasing plasticizer-to-polymer ratio. The incorporation of Bu-NENA increased the separation between the polyurethane chains and weakened interchain interactions. Meanwhile, the resulting increase in free volume enhanced segmental mobility, reducing the probability of stable hydrogen-bond formation between the urethane carbonyl and N-H groups. Consequently, the fraction of hydrogen-bonded carbonyl groups decreased as the plasticizer-to-polymer ratio increased.

**Figure 2:**
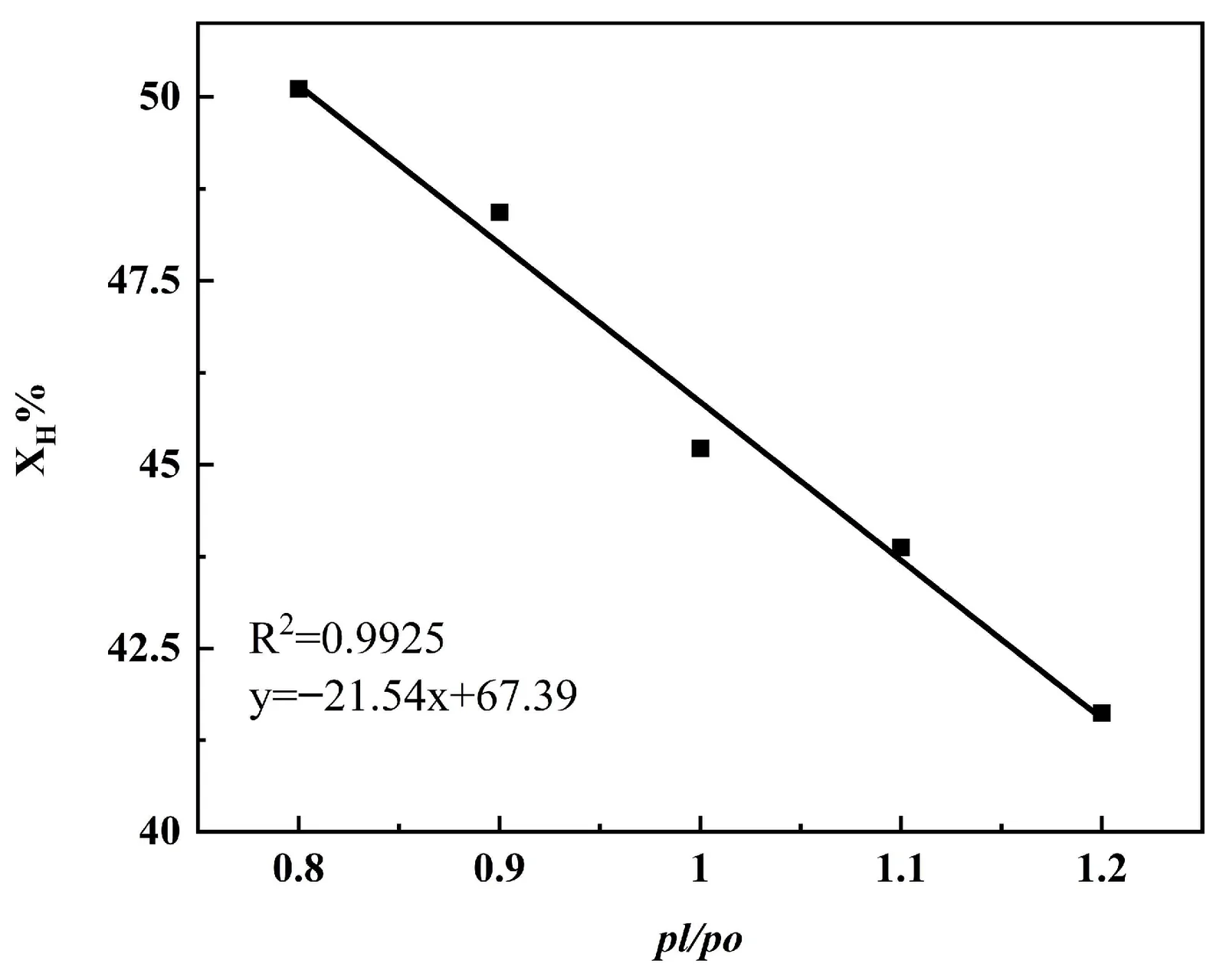
Linear fit of the fraction of hydrogen-bonded carbonyl groups as a function of the plasticizer-to-polymer ratio.

To quantify the relationship between the plasticizer-to-polymer ratio and the fraction of hydrogen-bonded carbonyl groups, *X_H_* was fitted as a function of the plasticizer-to-polymer ratio using linear regression. As shown in Figure 2, the following relationship was obtained:

*X_H_* = −21.54*P* + 67.39

*R*^2^ = 0.9925

where *P* denotes the plasticizer-to-polymer ratio. The fitting results reveal a strong negative linear correlation between *X_H_* and *P* over the range of 0.8–1.2. For every 0.1 increase in *P*, *X_H_* decreased by approximately 2.15 percentage points. Comparison of the integrated peak areas shows that *S*_1_ varied only slightly, whereas *S*_2_ increased from 6.54 to 10.05, corresponding to an increase of 53.67%. This result indicates that the decrease in *X_H_* was primarily associated with an increase in the free-carbonyl contribution. The presence of Bu-NENA within the polyurethane network increased the average interchain spacing and free volume, thereby reducing the spatial proximity of the urethane groups and the probability of stable hydrogen-bond formation between the carbonyl and N-H groups. Consequently, increasing the plasticizer-to-polymer ratio increased the free-carbonyl contribution and progressively weakened hydrogen-bonding association between the hard segments.

#### 3.1.2. Effect of the Plasticizer-to-Polymer Ratio on the Network Structure of iPCE Elastomers

**Figure 3:**
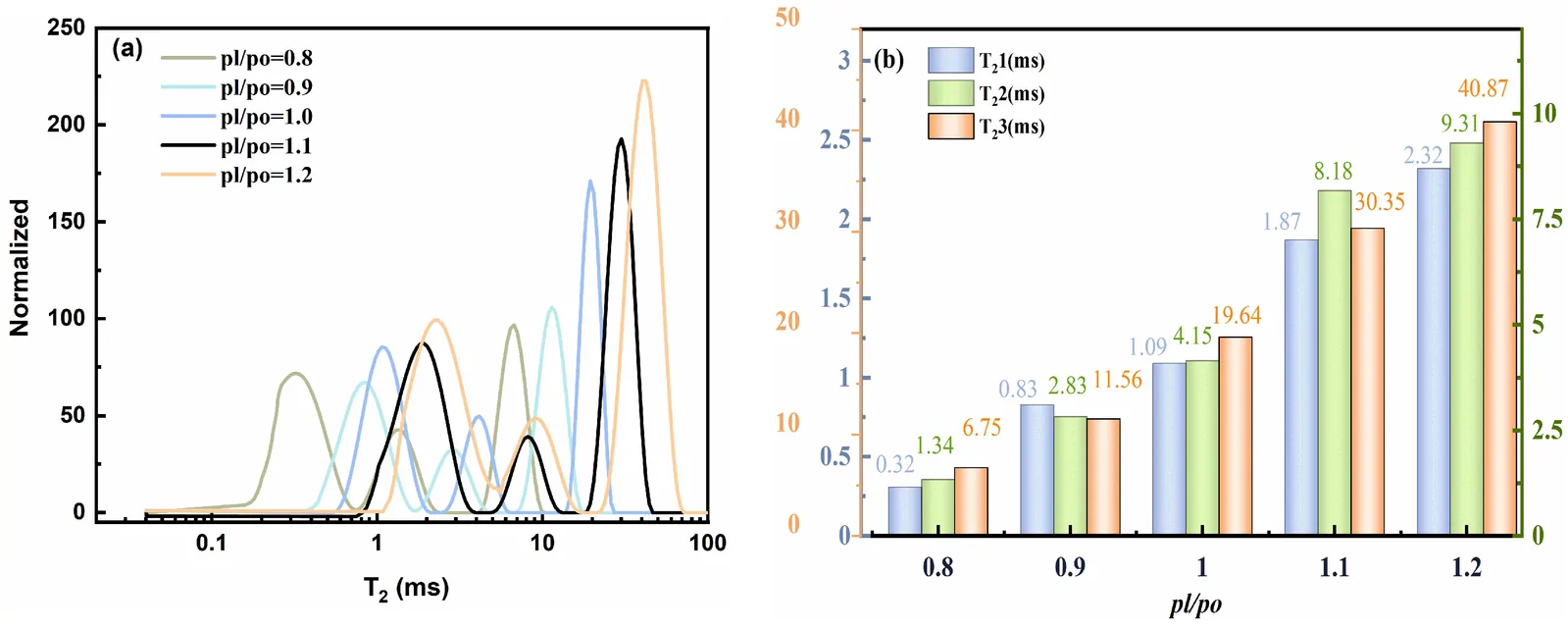
LF-NMR *T*_2_ relaxation characteristics of iPCE elastomers with different plasticizer-to-polymer ratios: (**a**) *T*_2_ relaxation-time distributions and (**b**) *T*_2_ values of the individual relaxation components.

Low-field nuclear magnetic resonance (LF-NMR) spectroscopy was used to examine the effects of the plasticizer-to-polymer ratio on the crosslinked network and segmental mobility of the iPCE elastomers. The network parameters derived from the transverse relaxation measurements are presented in Figure 3. The *T*_2_1 component is primarily attributed to restricted segments near the crosslinking points and within hard-segment-rich domains, whereas *T*_2_2 mainly represents the more mobile segments in the soft-segment-rich domains. The *T*_2_3 component is associated with highly mobile segments subject to weak network constraints or with unbound components.

As shown in Figure 3, the transverse relaxation times increased progressively with increasing plasticizer-to-polymer ratio, indicating enhanced segmental mobility and reduced motional constraints. Bu-NENA was physically distributed within the polyurethane network, where it increased the free volume and weakened interchain interactions and hydrogen-bonding association. These changes reduced the constraints imposed by the crosslinked network and facilitated segmental motion.

The apparent network parameters were obtained by fitting the transverse magnetization decay using the XLD model implemented in the crosslink-density analysis software:

y(t) = A exp[−t/T_2,A_ − 0.5qMrl t^2^] + B exp(−t/T_2,B_) + A_0_

where y(t) is the normalized NMR signal at time t; A is the signal fraction assigned to the network-constrained component, including contributions from chemical and physical crosslinks; T_2,A_ is the transverse relaxation time of the network-constrained component; q is the anisotropy factor of this component; Mrl is the residual dipolar moment below the glass-transition temperature; B and T_2,B_ are the signal fraction and transverse relaxation time, respectively, of the highly mobile component; and A_0_ is a constant offset used in the fitting procedure. The apparent crosslink density, *ν_e_*, was obtained from the network-constrained component using the calibration procedure implemented in the analysis software. The apparent average molecular weight between crosslinks, *M_c_*, was calculated according to the following equation:

*M_c_* = ρ/*ν_e_*

where ρ is the density of the elastomer (g·cm^−3^), *ν_e_* is the apparent crosslink density (mol·cm^−3^), and *M_c_* is the apparent average molecular weight between crosslinks (g·mol^−1^). Because the LF-NMR response includes contributions from permanent chemical crosslinks, hydrogen-bonding associations, chain entanglements, and other physical constraints, the calculated *ν_e_* values were interpreted as apparent network parameters rather than absolute chemical crosslink density [32].

Table 2 summarizes the effects of the plasticizer-to-polymer ratio and measurement temperature on the apparent crosslink density of the iPCE elastomers. At a given temperature, the apparent crosslink density decreased progressively with increasing plasticizer-to-polymer ratio, accompanied by an increase in the average molecular weight between crosslinks. These changes indicate a reduction in the effective constraints imposed by the network. Because Bu-NENA does not participate in the polyurethane crosslinking reaction, its incorporation dilutes the polymer network and weakens physical constraints arising from hydrogen bonding and chain entanglements, thereby enhancing segmental mobility. This interpretation is consistent with the observed increase in *T*_2_ relaxation times.

At a fixed plasticizer-to-polymer ratio, increasing the measurement temperature from 30 to 50 °C resulted in a lower apparent crosslink density and a higher average molecular weight between crosslinks. These changes reflect enhanced thermal motion of the polymer segments and reduced contributions from temperature-dependent physical crosslinks, including hydrogen-bonding associations. Thus, both an increased plasticizer-to-polymer ratio and an elevated temperature reduced the effective network constraints in the iPCE elastomers.

Because the PCL/PEG ratio, IPDI/N100 ratio, and NCO/OH molar ratio were maintained constant across all formulations, the decrease in the apparent crosslink density determined by LF-NMR should not be attributed simply to a change in the nominal stoichiometry of the polyurethane network. When considered together with the FTIR results, increasing the Bu-NENA content decreased the fraction of hydrogen-bonded carbonyl groups, prolonged the *T*_2_ relaxation times, reduced the apparent crosslink density, and increased the apparent average molar mass between crosslinks. These mutually consistent changes indicate that the physical plasticization induced by Bu-NENA weakened hydrogen-bonding associations and other effective physical constraints, thereby reducing the overall restriction imposed by the network on segmental motion. Previous research has similarly shown that plasticization can weaken effective interchain interactions and enhance molecular mobility in polyurethane-based elastomers [29]. By combining FTIR and LF-NMR analyses, the present study further establishes a direct correspondence between the reduction in hydrogen-bonding associations and the weakening of effective network constraints.

#### 3.1.3. Effect of the Plasticizer-to-Polymer Ratio on the Microphase Structure of iPCE Elastomers

**Figure 4:**
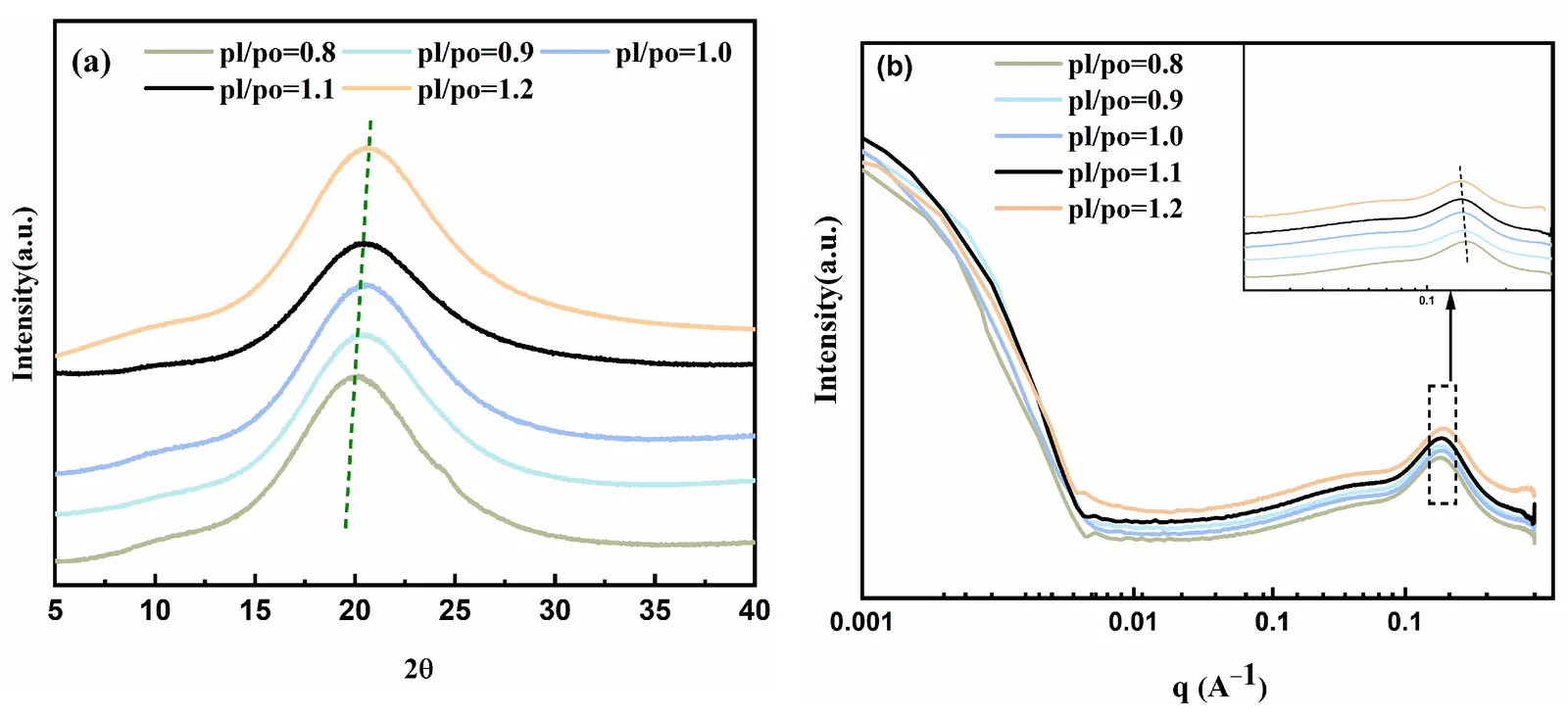
Microphase structures of iPCE elastomers with different plasticizer-to-polymer ratios: (**a**) WAXD patterns and (**b**) SAXS profiles.

Thermodynamic incompatibility between the soft and hard segments of polyurethane gives rise to a microphase-separated structure. The characteristic length scale and extent of microphase separation influence segmental mobility, network constraints, and mechanical properties. Because the incorporation of a plasticizer alters the free volume and intermolecular interactions, it can also affect segmental organization and domain morphology. The microphase structures of the iPCE elastomers prepared at different plasticizer-to-polymer ratios were therefore characterized by wide-angle X-ray diffraction (WAXD) and small-angle X-ray scattering (SAXS), as shown in Figure 4.

As shown in Figure 4a, all iPCE elastomers exhibited a broad amorphous halo near 2*θ* ≈ 20^∘^, with no discernible crystalline diffraction peaks. This result indicates that the samples were predominantly amorphous over the investigated range of plasticizer-to-polymer ratios.

As shown in Figure 4b, all samples exhibited a scattering maximum in the small-angle region, indicating periodic electron-density fluctuations associated with the microphase-separated morphology [33,34,35]. The characteristic domain spacing, *d*, was calculated from the position of the scattering maximum, *q_m_*, using *d* = 2*π*/*q_m_* [36]. The resulting structural parameters are summarized in Table 3. With increasing plasticizer-to-polymer ratio, the scattering maximum shifted toward lower *q* values and decreased in intensity, while *d* increased progressively. The shift of *q_m_* toward lower values, together with the corresponding increase in *d*, indicates an expansion of the characteristic periodicity of the microphase-separated structure.

Bu-NENA acts as a physical plasticizer and does not participate in the chemical crosslinking reaction. Its incorporation increases the free volume of the polyurethane network and may preferentially expand the soft-segment-rich domains and interfacial regions. Meanwhile, the weakened hydrogen-bonding association between urethane groups reduces hard-segment aggregation. Together, these effects increase the average spacing between hard-segment-rich domains and produce a less sharply defined domain contrast, consistent with the lower SAXS peak intensity observed at higher plasticizer-to-polymer ratios.

Previous studies on segmented polyurethanes have demonstrated that hydrogen-bond-mediated association between hard segments, together with the degree of compatibility and intermixing between the soft and hard segments, governs the characteristic length scale of the microphase-separated morphology [34,35]. In the present study, increasing the plasticizer-to-polymer ratio decreased both the fraction of hydrogen-bonded carbonyl groups and the apparent crosslink density. Concurrently, the characteristic SAXS peak shifted toward lower q values, corresponding to an increase in the characteristic spacing from 4.39 to 4.65 nm. Taking together, these results indicate that the effects of Bu-NENA are not limited to changes in local hydrogen-bonding interactions and segmental dynamics but are also associated with an increase in the average spacing between hard-segment-rich domains. Because Bu-NENA does not participate in the polyurethane curing reaction, it acts primarily as a physical plasticizer, enhancing segmental mobility and weakening the hydrogen-bonding associations that contribute to hard-segment aggregation. These changes are consistent with the observed increase in the characteristic length scale of the microphase-separated structure.

### 3.2. Effect of the Plasticizer-to-Polymer Ratio on the Mechanical Properties of iPCE Elastomers

**Figure 5:**
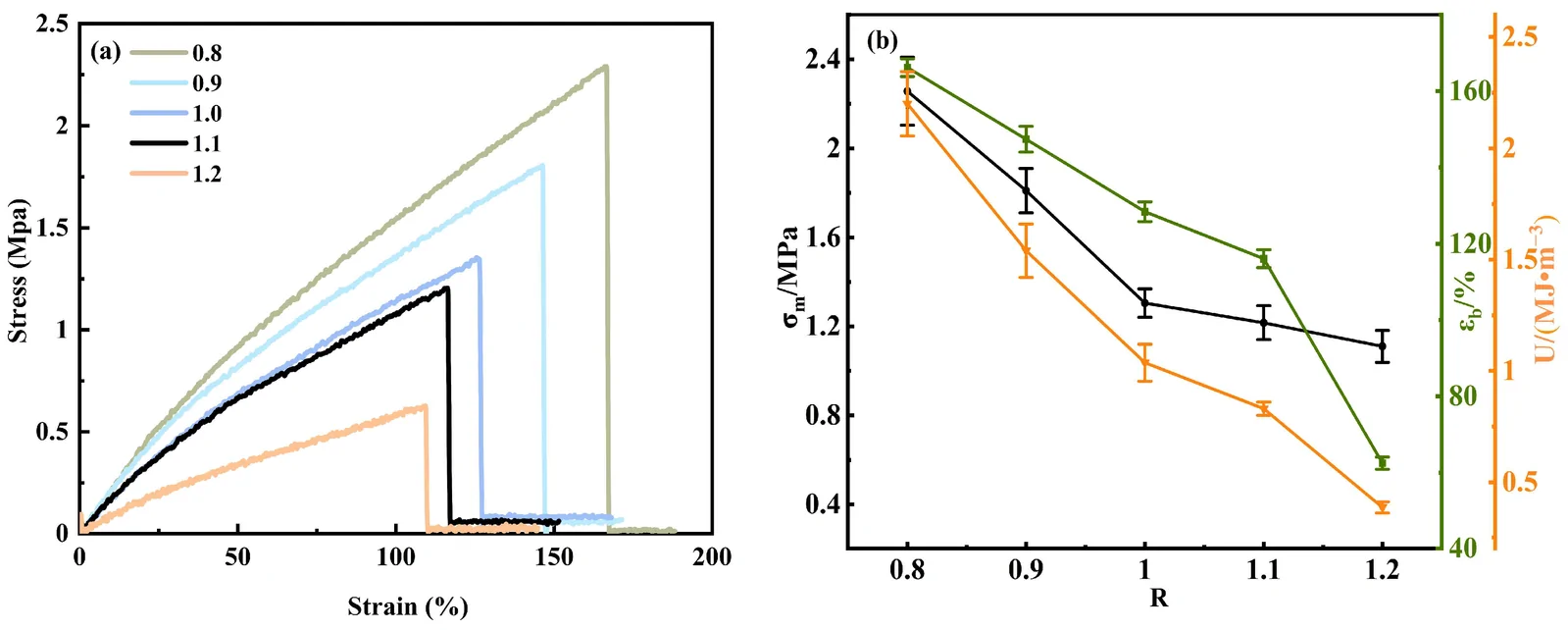
Effect of the plasticizer-to-polymer ratio on the mechanical properties of iPCE elastomers: (**a**) stress–strain curves and (**b**) variations in tensile properties with the plasticizer-to-polymer ratio.

Uniaxial tensile tests were performed to evaluate the effect of the plasticizer-to-polymer ratio on the mechanical properties of the iPCE elastomers. The resulting stress–strain curves are shown in Figure 5, and the tensile strength, elongation at break, and toughness are summarized in Table 4.

As shown in Figure 5, none of the iPCE elastomers exhibited a distinct yield point during tensile deformation. Both the tensile strength and elongation at break decreased progressively with increasing plasticizer-to-polymer ratio, indicating simultaneous reductions in load-bearing capacity and deformability. Bu-NENA acts as a physical plasticizer and does not participate in the chemical crosslinking reaction. Increasing its proportion dilutes the polyurethane network and weakens hydrogen-bonding associations, interchain interactions, and chain entanglements, consistent with the reduction in apparent crosslink density. Consequently, the fraction of network chains that effectively contribute to load transfer decreases. Under tensile loading, the weakened intermolecular interactions facilitate relative chain slippage and reduce the ability of the network to sustain continuous and stable stress transfer. Premature failure therefore occurs in regions with insufficient load-bearing capacity, resulting in concurrent decreases in tensile strength, elongation at break, and toughness.

The network structure and mechanical properties of the corresponding non-plasticized PEG/PCL-based polyurethane elastomer have previously been reported [37], providing a reference for evaluating the plasticization effect of Bu-NENA. Relative to the network characteristics reported for the non-plasticized system, the decreased fraction of hydrogen-bonded carbonyl groups and apparent crosslink density, together with the prolonged *T*_2_ relaxation times observed in the present study, indicate that Bu-NENA weakens hydrogen-bonding associations and other effective network constraints within the polyurethane network. As the plasticizer-to-polymer mass ratio increased from 0.8 to 1.2, the tensile strength, elongation at break, and toughness decreased simultaneously. In particular, the elongation at break decreased from 166.80% to 62.32%, while the toughness decreased from 2.16 to 0.39 MJ·m^−3^. Although the prolonged *T*_2_ relaxation times indicate enhanced segmental mobility, this increased mobility did not lead to improved extensibility. Instead, the concurrent decreases in the fraction of hydrogen-bonded carbonyl groups and apparent crosslink density, together with the increase in the characteristic SAXS spacing, indicate a progressive weakening of intermolecular associations and effective network constraints. These structural changes reduced the ability of the polyurethane network to transfer stress and maintain its integrity under large deformation, leading to failure at a lower strain. Therefore, excessive plasticization does not necessarily improve the extensibility of the elastomer when the network constraints required for effective stress transfer are substantially weakened.

### 3.3. Thermal Decomposition Behavior of Bu-NENA-Plasticized iPCE Elastomers

#### 3.3.1. TG-DTG Analysis of Bu-NENA-Plasticized iPCE Elastomers

**Figure 6:**
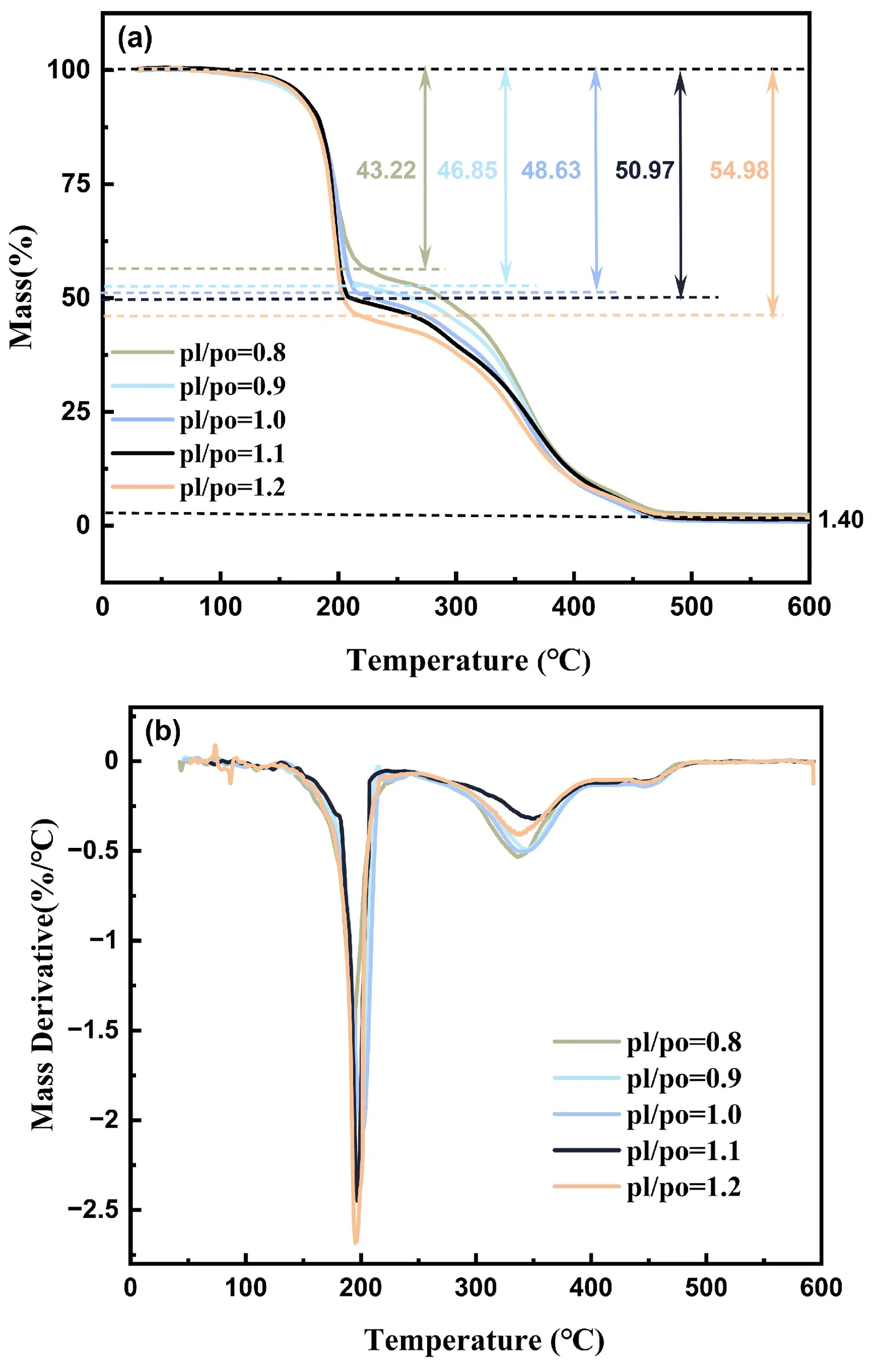
Thermal decomposition behavior of Bu-NENA-plasticized iPCE elastomers: (**a**) TG curves and (**b**) DTG curves.

Figure 6 presents the TG and DTG curves of the iPCE elastomers at different plasticizer-to-polymer ratios. All samples exhibited three principal mass-loss stages. Taking the elastomer with a plasticizer-to-polymer ratio of 0.8 as an example, the first stage occurred at 170–220 °C, with a DTG peak at approximately 200 °C and a mass loss of 43.22%. This value is close to the calculated Bu-NENA mass fraction of 44.44%, indicating that this stage is primarily associated with the decomposition of Bu-NENA. The second stage occurred at 250–400 °C, with a DTG peak at approximately 336 °C and a mass loss of 46.08%. This stage is mainly attributed to degradation of the polyurethane network, involving cleavage of the urethane linkages and decomposition of the ester- and ether-containing soft segments. The third stage occurred at 400–500 °C, with a DTG peak at approximately 445 °C and a mass loss of 8.30%, and is assigned to further decomposition of the macromolecular fragments and carbonaceous residues formed during the preceding stage.

Table 5 summarizes the mass loss associated with each decomposition stage for the iPCE elastomers at different plasticizer-to-polymer ratios. As *pl*/*po* increased from 0.8 to 1.2, the first-stage mass loss increased from 43.22% to 54.98%, closely following the increase in the theoretical mass fraction of Bu-NENA from 44.44% to 54.55%. In contrast, the combined mass loss in the second and third stages decreased from 54.38% to 44.55%, corresponding closely to the decrease in the theoretical polyurethane mass fraction from 55.56% to 45.45%. As shown in Table 6, the ratio ΔTG_1_/(ΔTG_2_ + ΔTG_3_) increased from 0.79 to 1.23, in close agreement with the increase in *pl*/*po*. These results support the assignment of the first mass-loss stage primarily to Bu-NENA decomposition, whereas the second and third stages are associated with degradation of the polyurethane network and further decomposition of the resulting residual structures, respectively. Thus, the relative mass losses of the three stages are broadly consistent with the proportions of Bu-NENA and polyurethane in the formulations.

#### 3.3.2. DSC Analysis

**Figure 7:**
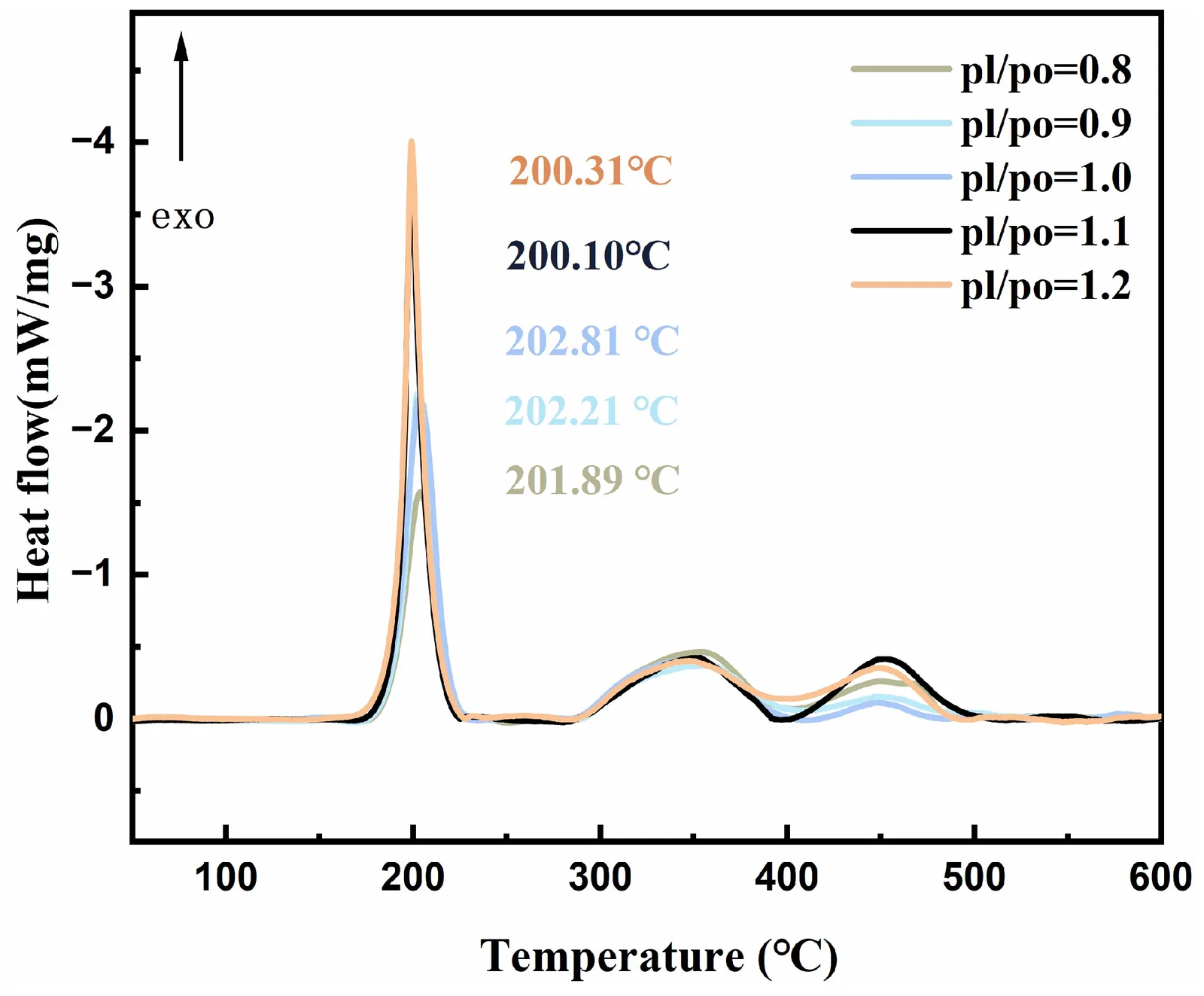
DSC curves of Bu-NENA-plasticized iPCE elastomers.

Figure 7 presents the DSC curves of the Bu-NENA-plasticized iPCE elastomers. All samples exhibited an endothermic peak near 200 °C, with peak temperatures ranging from 200.10 to 202.81 °C. Combined with the TG-DTG results, this endothermic event is assigned primarily to the first mass-loss stage dominated by Bu-NENA decomposition. As the plasticizer-to-polymer ratio increased, the endothermic peak became more pronounced, whereas its peak temperature changed only slightly. These results indicate that increasing the Bu-NENA content increased the magnitude of the thermal effect associated with this stage but had little influence on its characteristic temperature.

Over the temperature range of 300–500 °C, all samples exhibited broad, overlapping exothermic features, primarily associated with consecutive degradation of the polyurethane network and the resulting macromolecular fragments. The high-temperature exothermic region remained essentially unchanged across the investigated plasticizer-to-polymer ratios, and no additional thermal events were observed. These results indicate that varying the Bu-NENA content did not introduce a distinct major decomposition stage.

#### 3.3.3. Thermal Decomposition Mechanism of Bu-NENA Plasticized iPCE Elastomers

**Figure 8:**
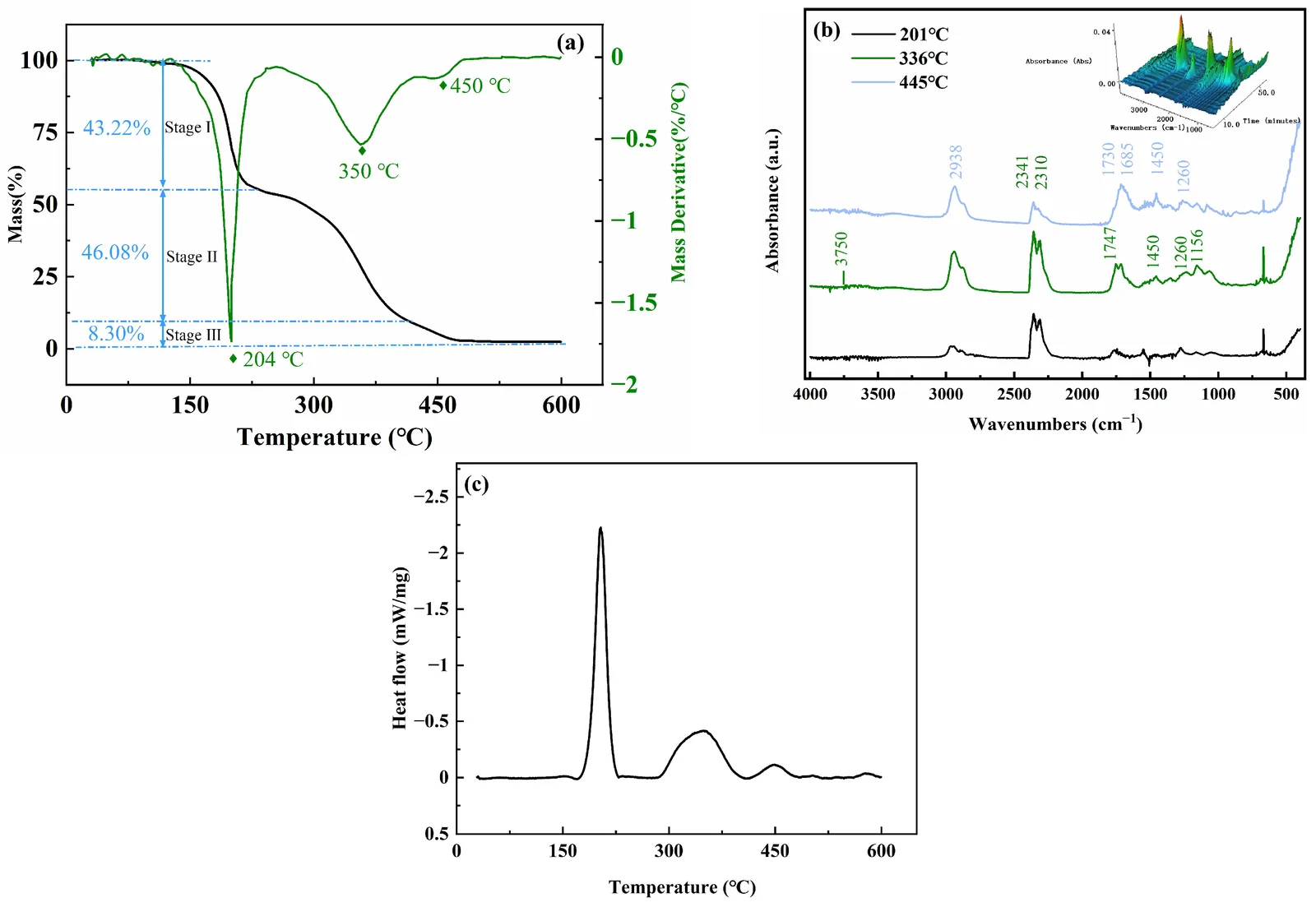
TG–DSC–FTIR analysis of Bu-NENA-plasticized iPCE elastomers: (**a**) TG and DTG curves and (**b**) FTIR curves and (**c**) DSC curve.

Figure 8 presents the TG-DSC-FTIR results for the Bu-NENA plasticized iPCE elastomers. As shown in Figure 8a, thermal decomposition proceeded through three principal mass-loss stages. The first stage was dominated by Bu-NENA decomposition, the second was primarily associated with degradation of the polyurethane network, and the third involved further decomposition of the soft-segment-derived macromolecular fragments and carbonaceous residues formed during the preceding stages. Figure 8b shows the FTIR spectra of the evolved gases recorded at the corresponding DTG peak temperatures, providing information on the gaseous products released during each decomposition stage. The characteristic thermal decomposition temperatures of the representative iPCE elastomer with a plasticizer-to-polymer ratio of 1.1, including T_5_%, T_10_%, and the maximum mass-loss-rate temperatures of the three decomposition stages, are summarized in Table 7.

The evolved-gas FTIR spectrum recorded at 201 °C (Figure 8b) exhibited characteristic CO_2_ absorption bands near 2341 and 2310 cm^−1^, together with bands attributable to aliphatic C-H groups and volatile oxygen-containing organic species. Given that the mass loss during the first stage was close to the theoretical mass fraction of Bu-NENA, this stage was assigned primarily to the thermal decomposition of Bu-NENA [38]. The decomposition is initiated by homolytic cleavage of the O-NO_2_ bonds in the nitrate ester groups, generating alkoxy and NO_2_ radicals [38,39]. Subsequent fragmentation and secondary radical reactions produce CO_2_ and other low-molecular-weight gaseous products.

At 336 °C, the evolved-gas FTIR spectrum contained a substantially greater number of absorption bands. The band near 3750 cm^−1^ was assigned to O-H stretching, while that at 1747 cm^−1^ was attributed to C=O stretching in carbonyl-containing species. The band near 1450 cm^−1^ corresponded to aliphatic C-H bending, and those near 1260 and 1156 cm^−1^ were associated with C-O-C and C-O stretching vibrations, respectively [40]. These spectral features are consistent with consecutive degradation of the urethane linkages and the polyester- and polyether-based soft segments, producing CO_2_ and various volatile products containing carbonyl, ether, and hydroxyl functionalities [41]. This stage therefore corresponds primarily to the principal mass loss of the polyurethane network.

At 445 °C, the band at 2938 cm^−1^ was assigned to aliphatic C-H stretching, whereas the bands at 1730 and 1685 cm^−1^ were attributed to C=O stretching vibrations of carbonyl-containing species in different chemical environments [30,42]. Absorption bands associated with CO_2_ and other oxygen-containing species were also observed [43]. These features indicate that the macromolecular fragments formed during the preceding stage underwent further fragmentation and secondary reactions, including decomposition of the carbonyl-containing structures and decarboxylation, producing CO_2_, hydrocarbons, and low-molecular-weight carbonyl compounds. This stage was therefore assigned primarily to further decomposition of the residual polymer structures and carbonaceous intermediates [44].

The Bu-NENA plasticized iPCE elastomers underwent three consecutive thermal decomposition stages: Bu-NENA decomposition, degradation of the polyurethane network, and further decomposition of the residual structures at elevated temperatures. The principal processes associated with each stage are summarized as follows:

Bu-NENA decomposition, involving initial O-NO_2_ bond cleavage followed by secondary radical reactions that produce CO_2_ and other low-molecular-weight volatile products.

Cleavage of the urethane linkages and degradation of the polyester- and polyether-based soft segments, corresponding to the principal mass loss of the polyurethane network.

Further fragmentation and secondary decomposition of the residual macromolecular structures formed during the preceding stage.

In contrast to the pronounced changes in mechanical performance, the principal decomposition stages and their characteristic temperatures remained nearly unchanged as the plasticizer-to-polymer ratio varied. Increasing the Bu-NENA content mainly altered the relative mass loss associated with each stage. This difference indicates that the mechanical response was highly sensitive to changes in intermolecular interactions and effective network constraints, whereas the thermal decomposition behavior was governed primarily by the relative proportions and intrinsic decomposition characteristics of Bu-NENA and the polyurethane matrix.

## 4. Conclusions

Increasing the plasticizer-to-polymer ratio enhanced the physical plasticization of the polyurethane network by Bu-NENA, as evidenced by increased free volume, weakened hydrogen-bonding associations, reduced apparent crosslink density, and enhanced segmental mobility. Concurrently, the characteristic spacing between the soft- and hard-segment-rich domains increased, and the SAXS peak intensity decreased, whereas no discernible crystallization was observed. These structural changes impaired stress transfer and deformation stability within the polyurethane network, resulting in progressive decreases in tensile strength, elongation at break, and toughness. Thus, excess Bu-NENA not only reduced the load-bearing capacity of the elastomers but also promoted premature network failure under large deformation.

The Bu-NENA-plasticized iPCE elastomers underwent three consecutive thermal decomposition stages: Bu-NENA decomposition, degradation of the polyurethane network, and further decomposition of the residual structures at elevated temperatures. Varying the plasticizer-to-polymer ratio primarily altered the relative mass loss associated with each stage but had little effect on the principal decomposition stages or their characteristic temperatures. Overall, the plasticizer-to-polymer ratio affected the mechanical response of the iPCE elastomers mainly by modifying intermolecular interactions and effective network constraints, whereas its influence on thermal decomposition was primarily associated with changes in the relative proportions of Bu-NENA and polyurethane.

Within investigations, a plasticizer-to-polymer ratio of 1.0 provided the most appropriate balance between physical plasticization and mechanical performance. At this ratio, the elastomer exhibited enhanced segmental mobility and reduced intermolecular constraints while retaining a tensile strength of 1.35 MPa, an elongation at break of 126.49%, and a toughness of 1.01 MJ·m^−3^. Ratios of 0.8 and 0.9 provided better mechanical properties but comparatively limited plasticization, whereas further increasing the ratio to 1.1 or 1.2 caused a marked deterioration in mechanical performance, particularly in elongation at break and toughness. Compared with conventional inert ester plasticizers, Bu-NENA offers the advantage of contributing to the energetic output of the formulation while providing physical plasticization. However, its nitrate ester functionality introduces a distinct low-temperature decomposition stage at approximately 199 °C, and excessive incorporation substantially weakens the mechanical integrity of the polyurethane network.

## Figures and Tables

**Table 1:** Quantities of raw materials used for representative formulation with a plasticizer-to-polymer ratio of 1.0.

Raw Material	*m* (g)
PCL	3.80
PEG	1.90
IPDI	2.82
N100	2.38
Catalyst	0.05
Bu-NENA	10.95

**Table 2:** Fraction groups in iPCE elastomers with different plasticizer-to-polymer ratios.

*pl/po*	Peak Area		*X_H_*%
	*S*_1_ (1690 cm^−1^)	*S*_2_ (1725 cm^−1^)	
0.8	6.57	6.54	50.11
0.9	6.79	7.24	48.43
1.0	7.38	8.94	45.22
1.1	7.69	9.84	43.87
1.2	7.16	10.05	41.62

**Note:** *S*_1_ and *S*_2_ denote the integrated peak areas of the hydrogen-bonded and free carbonyl groups at 1690 and 1725 cm^−1^, respectively. *X_H_* represents the fraction of hydrogen-bonded carbonyl groups.

**Table 3:** Effects of the Plasticizer-to-Polymer Ratio and Temperature on the Crosslink Density of iPCE Elastomers.

*pl/po*	30 °C		40 °C		50 °C	
	*v_e_* (_10_^−4^ mol/cm^−3^)	*M_c_* (_10_^3^ g/mol)	*v_e_* (_10_^−4^ mol/cm^−3^)	*M_c_* (_10_^3^ g/mol)	*v_e_* (_10_^−4^ mol/cm^−3^)	*M_c_* (_10_^3^ g/mol)
0.8	4.32	2.03	3.99	2.06	3.71	2.14
0.9	4.21	2.06	3.91	2.10	3.63	2.17
1.0	4.09	2.10	3.79	2.14	3.52	2.21
1.1	3.94	2.13	3.65	2.17	3.39	2.24
1.2	3.81	2.15	3.54	2.19	3.29	2.26

**Table 4:** Structural parameters of iPCE elastomers with different plasticizer-to-polymer ratios.

*pl/po*	*q_m_* (nm^−1^)	d (nm)
0.8	1.43	4.39
0.9	1.40	4.49
1.0	1.37	4.59
1.1	1.36	4.62
1.2	1.35	4.65

***Note:*** *pl/po denotes the plasticizer-to-polymer mass ratio.*

**Table 5:** Tensile strength and elongation at break of iPCE elastomer films with different plasticizer-to-polymer ratios.

*pl/po*	*σ_m_* (MPa)	*ε_b_* (%)	*U* (MJ⋅m^−3^)
0.8	2.29	166.80	2.16
0.9	1.81	146.37	1.54
1.0	1.35	126.49	1.01
1.1	1.20	116.28	0.83
1.2	1.09	62.32	0.39

**Table 6:** Mass Loss during Each Stage of Thermal Decomposition.

*pl/po*	ΔTG_1_	ΔTG_2_	ΔTG_3_	ΔTG_1_/(ΔTG_2_ + ΔTG_3_)
0.8	43.22	46.08	8.30	0.79
0.9	46.85	43.67	7.88	0.90
1.0	48.63	42.76	7.14	0.98
1.1	50.97	41.10	5.97	1.08
1.2	54.98	39.14	5.41	1.23

**Note:** ΔTG_1_, ΔTG_2_ and ΔTG_3_ denote the mass losses during the first, second, and third decomposition stages, respectively.

**Table 7:** Characteristic thermal decomposition temperatures of the iPCE elastomer.

TG 5% (°C)	TG 10% (°C)	TG 1,max (°C)	TG 2,max (°C)	TG 3,max (°C)
164.78	180.22	200.31	336.25	445.37

## Data Availability

The data supporting the findings of this study are available from the corresponding author upon reasonable request.

## Supplementary Materials

The following supporting information can be downloaded at: https://www.mdpi.com/article/10.3390/polym18192402/s1, Figure S1: Dimensions of the narrow parallel section of the dumbbell-shaped specimen used for tensile testing.
