# Supplementary material for: Effect of the Plasticizer-to-Polymer Ratio on the Properties of In Situ-Prepared PCL/PEG-Based Polyurethane Elastomers
Source: Polymers (Basel). 2026 Oct 1;18(19):2402. doi: 10.3390/polym18192402 (PMC13667257; doi:10.3390/polym18192402)
Supplement: Supplementary file 1 [file polymers-18-02402-s001.zip › polymers-4538045-supplementary.pdf]

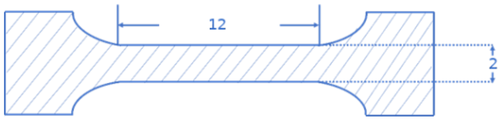

Figure S1. Dimensions of the narrow parallel section of the dumbbell-shaped specimen used for tensile testing.
